# Recent measles outbreak in unvaccinated children in Ohio: cause and causality – a correspondence

**DOI:** 10.1097/JS9.0000000000000215

**Published:** 2023-02-16

**Authors:** Olivier Uwishema, Heeba Anis, Sarah El Kassem, Ali E. Hamitoglu, Dina Essayli, Abubakar Nazir

**Affiliations:** a Oli Health Magazine Organization, Research, and Education, Kigali, Rwanda; b Clinton Global Initiative University, New York, USA; c Faculty of Medicine, Karadeniz Technical University, Trabzon; d Namik Kemal University Faculty of Medicine, Merkez/Tekirdag, Turkey; e Faculty of Medicine, Beirut Arab University, Beirut; f Lebanese University – Faculty of Medicine, Hadat Campus, Lebanon; g Deccan College of Medical Sciences, Hyderabad, Telangana, India; h Department of Medicine, King Edward Medical University, Lahore, Pakistan

Highlights

- Measles is a highly communicable viral disease that belongs to the paramyxovirus family and is spread through airborne and direct contact. Nonimmune individuals are at a very high risk of being infected and potentially causing outbreaks.
- Guardians who are cautious about vaccinating their children with vaccines officially declared by the health sector in the state of Ohio will most likely postpone their vaccination schedule and create outbreaks of diseases that are vaccine-preventable.
- Outbreaks of measles can spawn epidemics that result in death, especially in nonvaccinated children. As a result, vaccination against measles is one of the precautionary measures that help in preventing disease diagnosis, progression and complications.

*Dear Editor*,

One of the most notorious viral illnesses affecting the paediatric age group worldwide is measles. A strong association was found between the recent outbreak of measles in Ohio and the disruption of vaccination activities in the region. It is therefore of paramount importance that culprits of this havoc, like vaccine hesitancy, incomplete immunisation, and prevalence of misinformation regarding the disease and vaccine efficacy, should be addressed by stakeholders through effective communication strategies to prevent future morbidity and mortality by the upheaval of such highly contagious disease.

Several countries, in addition to the United States (US), have shown a rise in hesitancy in administering vaccines which eventually led to under-vaccination. Globally, in the first 2 months of 2022, the number of reported measles cases increased by 79% compared to the same period in 2021. Twenty-three million children missed out on all basic childhood vaccines in 2020. The highest number of unvaccinated children since 2009^1^. A study that evaluated population-level measles in the US informs us that currently 9,145,026 children (13.1%) are estimated to be vulnerable to measles^2^. Furthermore, with pandemic-level vaccination amounts, 15,165,221 children (21.7%) will be susceptible to measles if no steps are taken to organise catch-up vaccination campaigns. Columbus Public Health has announced that since the beginning of the measles outbreak in Ohio, at least 59 children were reported to be infected, such that 56 out of 59 children were unvaccinated against measles^3^. Nowadays, measles vaccination in the US is high, where 90.8% of children receive no less than one measles, mumps and rubella vaccine^4^. Although national vaccination coverage remains high, sustained transmission can last in communities where vaccine hesitancy is present, noting that in 2019 the US experienced its highest number of measles cases since 1992^4^.

It is worth mentioning that a significant percentage of measles cases occurring during outbreaks in the US after the elimination period are deliberately unvaccinated. Therefore, reappearing outbreaks of measles, which is a vaccine-preventable disease, are highly associated with intentional vaccine hesitancy^5^.

Measles can be detected through several precise methods. PCR is one of the methods for detecting viral RNA, depending on nasal or oral or urine samples. Enzyme-linked immunosorbent assay is another technique based on serum samples that allows the detection of immunoglobulin M and immunoglobulin G antibodies in the exanthematous period and convalescent period, respectively^6^. Another proper method for measles surveillance is the rapid diagnostic test using blood, serum or oral fluid samples^7^. There is no specific antiviral treatment for measles where it is based on treating the symptoms of the virus, but vaccination is considered a major prophylactic method, where herd immunity can be done through vaccinating children at 15 months of age and above until the percentage of vaccinated children reach 85–95%^7^.

Measles is one of the communicable diseases that are highly targeted for worldwide elimination; however, although the goal of measles elimination is found in almost every part of the world, maintained elimination has not been achieved yet. Measles elimination strategies include improving the delivery of national immunisation services worldwide and sustaining a peak in vaccination coverage to save the lives of almost every child at risk^8^. Universal health coverage can be supported by the implementation of surveillance and other measles activities, such as case management as an element in the primary health care systems. Moreover, community and political engagement and improvement of measles goals accountability and identifying gaps are essential steps. Furthermore, catch-up vaccination remains important for people who missed their second dose of the vaccine. An essential strategy is to be prepared to respond to an outbreak in case it happens with early detection and thus diminishing morbidity and mortality as much as possible^9^. For adequate vaccination, the supply of measles vaccines should be available, maintained and monitored. Also, to cross barriers, research should always be sustained^10^.

## Ethical approval

Not applicable.

## Sources of funding

Not applicable.

## Author contribution

O.U.: conceptualization, project administration, and writing – review and designing. All the authors were involved in manuscript writing, data collection and assembly, and the final approval of the manuscript.

## Conflicts of interest disclosure

There are no conflicts of interest.

## Guarantor

A. Nazir, Oli Health Magazine and Organization, Kigali, Rwanda. E-mail: abu07909@gmail.com.

## References

[1] UNICEF. Measles cases are spiking. UNICEF; May 2022. Accessed 14 December 2022. https://www.unicef.org/stories/measles-cases-spiking-globally

[2] Gambrell A Sundaram M Bednarczyk RA . Estimating the number of US children susceptible to measles resulting from COVID-19-related vaccination coverage declines. Vaccine 2022;40:4574–9. 35728989 10.1016/j.vaccine.2022.06.033 PMC9197781

[3] Hübschen JM Gouandjika-Vasilache I Dina J . Measles. Lancet 2022;399:678–690. 35093206 10.1016/S0140-6736(21)02004-3

[4] Nelson R . US measles outbreak concentrated among unvaccinated children. Lancet Infect Dis 2019;19:248. 30833064 10.1016/S1473-3099(19)30075-1 PMC7129894

[5] Phadke VK Bednarczyk RA Salmon DA . Association between vaccine refusal and vaccine-preventable diseases in the United States: a review of measles and pertussis. JAMA 2016;315:1149–58; Erratum in: JAMA. 2016 May 17;315(19):2125. 26978210 10.1001/jama.2016.1353 PMC5007135

[6] Xavier AR . Clinical, laboratorial diagnosis and prophylaxis of measles in Brazil. J Bras Patol Med Lab 2019;55. Accessed 17 December 2022. https://www.scielo.br/j/jbpml/a/d4HfzvcFGZ75SYHL9ZZhkkt/?format=html&lang=en

[7] Uwishema O Adriano LF Torbati T . Measles crisis in Africa amidst the COVID-19 pandemic: delayed measles vaccine administration may cause a measles outbreak in Africa. J Med Virol 2021;93:5697–9. 34181289 10.1002/jmv.27150

[8] Centres for Disease Control and Prevention. What CDC is doing about global measles and rubella. Centres for Disease Control and Prevention; 2022. Accessed 16 December 2022. https://www.cdc.gov/globalhealth/measles/what/index.html

[9] World Health Organization. Measles and rubella strategic framework 2021–2030. World Health Organization; 2020.

[10] Berjaoui C Tabassum S Sabuncu Ö . Measles outbreak in Zimbabwe: an urgent rising concern. Ann Med Surg (Lond) 2022;82:104613. 36124222 10.1016/j.amsu.2022.104613 PMC9482105

